# A Rare Case of Co-occurring Ménière’s Disease and Red Ear Syndrome

**DOI:** 10.7759/cureus.108604

**Published:** 2026-05-10

**Authors:** Edie L Sperling, Mohammed E Elsalanty

**Affiliations:** 1 Medical Anatomical Sciences, Western University of Health Sciences, Lebanon, USA; 2 Medical Anatomical Sciences, Western University of Health Sciences, Pomona, USA

**Keywords:** clinical case report, ménière's disease, red ear syndrome, tinnitus, vertigo

## Abstract

Ménière’s disease (endolymphatic hydrops) is a multifactorial syndrome affecting the inner ear, typically distinguished by attacks of variable frequency which include vertigo, aural fullness, tinnitus, and fluctuating hearing loss. Red ear syndrome is characterized by paroxysmal erythema of the external ear, either unilaterally or bilaterally, with pain, burning sensation, and/or swelling. Both conditions have complex and heterogeneous etiologies. Ménière’s disease and red ear syndrome have not previously been associated in the literature, yet they can have overlapping characteristics. This case describes the co-occurrence of Ménière’s disease and red ear syndrome in the right ear of a 43-year-old female patient. It highlights the potential association between the two conditions and proposes possible pathophysiological mechanisms linking them, including atypical migraine, autonomic dysregulation, vascular permeability, and hormonal factors.

## Introduction

Ménière’s disease (endolymphatic hydrops) is a multifactorial syndrome affecting the inner ear, typically distinguished by attacks of variable frequency which include episodic vertigo, aural fullness, tinnitus, and fluctuating hearing loss, often leading to permanent sensorineural hearing loss [1]. Prevalence is estimated at 190 per 100,000 persons, higher in females and among those of European descent compared to Asian, African, or Native American populations [2,3]. While it typically begins in one ear, it may eventually affect both ears in up to 45% of cases [3]. Diagnosis is often delayed due to the heterogeneity of patient presentations, the ambiguity of potential predisposing factors, and multiple potential pathophysiological pathways [1,4]. Ménière’s disease has been hypothesized to have multiple subtypes, some of which may still be unidentified [3]. Studies propose several contributory factors, including genetic factors (~10%), pre-existing respiratory or food allergies, autoimmune conditions, hormonal factors, viral infections, and autoinflammation [3]. Furthermore, migraines, vestibular migraines, benign paroxysmal positional vertigo, anatomical variants of the inner ear, trauma, psychological stress, and anxiety have all been identified as comorbid with Ménière’s disease [3]. An association between Ménière’s disease and red ear syndrome is not currently described in the literature.

Red ear syndrome is a rare condition first described in 1994, characterized by paroxysmal erythema of the external ear, either unilaterally or bilaterally, and is commonly associated with pain, burning sensation, and/or swelling [5,6]. Erythema and pain may spread to the cheek or neck [5]. Episodes typically last from several seconds to several hours and can be spontaneous or triggered by touch or pressure to the ear, mastication and oral/perioral functions, heat or temperature changes, exertion, head or neck movements, and stress [6,7]. Episodes range in frequency from monthly to multiple times per day, and most patients have both spontaneous and triggered episodes [8]. The pathophysiology of red ear syndrome is variable, with a range of potential etiologies or comorbidities, including migraine, trigeminal autonomic cephalalgias, vasculitis, C2/3 radiculopathy, cervical spine arachnoiditis or spondylosis, trigeminal neuralgia, and cranial autonomic symptoms [6-8].

Ménière’s disease and red ear syndrome have not previously been associated in the literature, yet they have potentially overlapping symptoms and characteristics. This case presents a 43-year-old female diagnosed with right-sided Ménière’s disease with a history of right-sided red ear syndrome since childhood, with no history of migraine.

## Case presentation

A 43-year-old female presented to the otolaryngology clinic with a five-year history of episodic aural fullness, tinnitus, hearing loss, vestibular impairment with loss of balance and feeling of "the floor moving and dropping," and attacks of nausea, vomiting, and unprovoked drop attacks (sudden falls without loss of consciousness). Onset was insidious with worsening symptoms for the first two years, followed by milder symptoms and a frequency of episodes reduced to two to three times per month. The episodes lasted for up to several hours. The patient did not have a history of migraine, including International Classification of Headache Disorders (ICHD-3) vestibular migraine diagnostic criteria of unilateral moderate or severe throbbing headache, visual disturbances/aura, photophobia, or phonophobia [9]. The patient denied having any prodromic symptoms preceding episodes, but reported a feeling of imbalance and vertigo for approximately 48 h following attacks. She reported a single incident of benign paroxysmal positional vertigo in 2021, which resolved fully after she self-performed the Epley maneuver. Patient’s medication history included a 10-year sertraline prescription for general anxiety, with no side effects. The patient also had a history of benign pre-ventricular contractions and an eating disorder, but no other health conditions prior to the start of symptoms. She had no history of head trauma. She reported that her occupation as a professor caused moderate stress, which worsens symptom frequency and severity. A history of typical migraine on the maternal side was reported.

The patient's symptoms commenced at the age of 38 years. Initial otolaryngologic exam yielded a diagnosis of vestibular migraine, which was attributed to hormonal changes associated with perimenopause. The hearing test was normal. A three-month prescription of amitriptyline did not improve symptoms, and no further follow-up was done. At the visit, the patient was prescribed ondansetron for nausea, which she continues to take as needed for symptom management. She declined a food elimination diet or dietary changes, which were recommended for migraines (e.g., removal of chocolate, aged cheese), due to a history of eating disorders. The patient was already limiting alcohol use to once or twice per month due to symptom aggravation. One year later, she was prescribed promethazine (12.5 mg) by her primary care doctor for use during episodes and sought referral to vestibular rehabilitation. She completed six visits with a vestibular physical therapist and reported that the sessions worsened symptoms during and after the session for approximately 48 h with no noticeable long-term changes.

On examination, after five years of symptoms, the patient was well oriented to time, place, and person. External examination revealed a normal pinna. Otoscopic examination revealed mild scarring from bilateral myringotomy tubes placed twice in each ear during childhood. Neurological examination revealed positive Hoffman’s sign bilaterally, which can be a sign of upper motor neuron disorder but can also commonly be a normal finding, which it was determined to be in this patient due to no other upper motor neuron signs. Impedance audiometry tests were normal.

**Figure 1:**
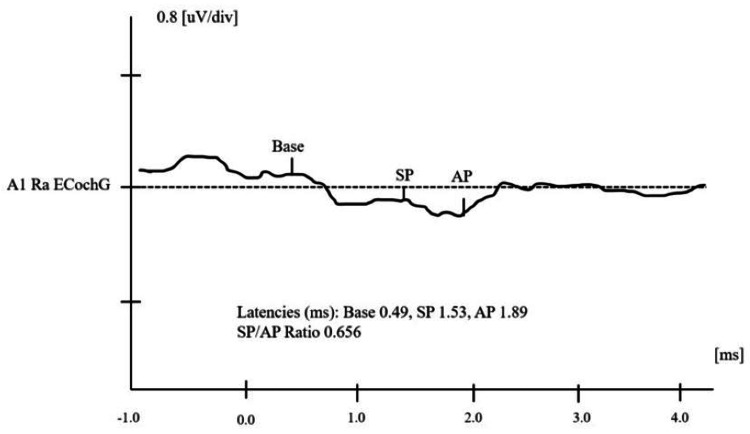
Electrocochleography results, SP/AP ratio >0.5 indicative of endolymphatic hydrops. AP: action potential; SP: summating potential

The patient was referred to audiology for electrocochleography; video-nystagmography (VNG), including gaze, smooth pursuit, oscillating tracking, random saccades, positional, and caloric tests; Hallpike testing; full-field optokinetic reflex (OKR); and hearing tests. Electrocochleography was consistent with a diagnosis of Ménière’s disease/endolymphatic hydrops in the right ear (Figure 1). The patient also had a left-eye, right-beating horizontal nystagmus with gaze to the left. Hearing tests were +10 decibels in the right ear and +5 decibels in the left ear, both in the normal range. The patient continues to report hearing loss and tinnitus in the right ear during attacks, and tinnitus intermittently throughout the day, every day. She was prescribed 24 mg of betahistine twice daily, and since beginning the medication two months ago, she has reported a reduced frequency of tinnitus and no further drop attacks to date.

**Figure 2:**
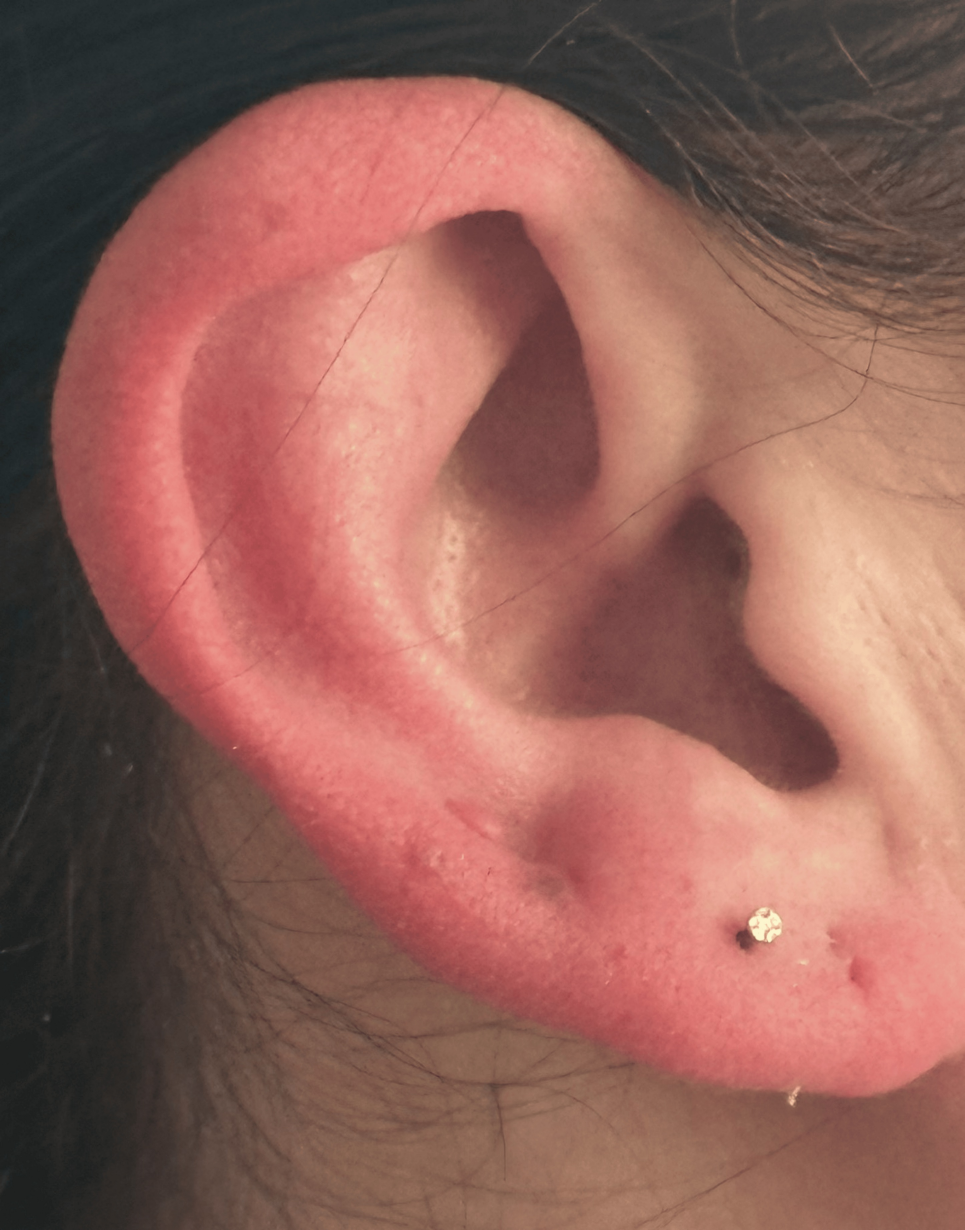
Erythematous episode of red ear syndrome in patient’s right pinna and earlobe.

The patient further reported episodic erythema of the right ear occurring since childhood, associated with a burning sensation and mild pain (2 out of 10 on visual analog scale), with erythema spreading to the ipsilateral cheek. Symptoms are aggravated by touching or rubbing the ear, but also occur spontaneously. Red ear symptoms did not coincide with Ménière’s attacks. Episodes occurred approximately 1-5 times per week and lasted for 30-45 min (Figure 2). The patient denies any burning sensation in the hands or feet or pain in other locations. The patient also had no family history of similar conditions. Complete blood count, metabolic panel, erythrocyte sedimentation rate, anti-nuclear antibody, and type II collagen antibody labs were normal.

Erythema symptoms were relieved by cold packs and over-the-counter anti-inflammatories (Aleve). When the patient was asked to rub her right pinna, a bright erythema was noted beginning after 30 s, with flushing spreading to the cheek after approximately 1 min (Figure 2). Signs and symptoms were consistent with a diagnosis of red ear syndrome.

## Discussion

The co-occurrence of Ménière’s disease and red ear syndrome has not previously been described in the literature, but the conditions have overlapping characteristics. No definitive etiology has been described for either Ménière’s disease or red ear syndrome, but migraine disorders and autonomic dysfunction have been observed in both conditions [3,6-8]. Additional congruent mechanisms may include an autoimmune genesis and/or inflammatory pathways [3,6-8]. In relation to the current patient, we describe several pathways below that could hypothetically influence the pathophysiology of both Ménière’s disease and red ear syndrome in a single patient. Further research into both conditions is ongoing, and results may assist in identifying etiologies and targeting improved treatment options.

Migraine

Migraine has been strongly linked to both Ménière’s disease and red ear syndrome [10,11]. Migraine is approximately twice as common in patients with Ménière’s disease [10]; red ear syndrome epidemiological data are scarce, but migraine is often associated with red ear syndrome in the case studies [11,12]. Aural pressure, fluctuating hearing loss, and tinnitus can also occur in vestibular migraine, without the permanent loss of hearing typically associated with Ménière’s disease. Migraine symptoms, such as headache and photophobia, were commonly reported during Ménière’s attacks [9]. International Classification of Headache Disorders (ICHD) diagnostic criteria for vestibular migraines include a current or past history of migraine with or without aura, which this patient denied [9]. Also absent were other necessary diagnostic symptoms of vestibular migraine (unilateral headache, pulsating headache, moderate or severe headache pain, headache aggravated by routine physical activity, photophobia or phonophobia, or visual aura without pain) [9]. Therefore, the patient’s presentation did not fit the ICHD criteria for migraine or vestibular migraine.

Patients with atypical migraine may not fulfill ICHD-3 diagnostic criteria, but present with migraine-like symptoms and/or respond to migraine treatment [13]. These symptoms can include vestibular symptoms without pain and autonomic symptoms [13]. Early-onset red ear syndrome, as in the case of this patient, is more commonly associated with migraine than cervical (C3) pathology, which is unlikely in children [8]. It has been proposed that migraine-associated red ear syndrome may result from trigemino-vascular activation, causing the release of vasodilator substances (e.g., substance P); trigeminal and vascular involvement helps explain the extension of ear pain beyond the trigeminal (V2) sensory innervation area [8].

The patient, therefore, may have atypical migraine without pain or aura, associated with Ménière’s disease and red ear syndrome, occurring on the right side. There is a maternal history of (typical) migraine in her family, which lends additional credence to this theory. However, patient symptoms responded well to betahistine - typically prescribed for Ménière’s disease - and not at all to amitriptyline, which is typically prescribed for migraine, leaving a migraine association unclear.

Autonomic dysregulation

Autonomic dysregulation may be a common denominator for Ménière’s disease and red ear syndrome [14,15]. One study reported that episodes of Ménière’s disease were associated with increased sympathetic activity and decreased parasympathetic activity on the affected side, beginning prior to the attack and returning to normal after the attack [14]. Limited sympathetic projections exist to the inner ear, so sympathetic pathways were not thought to play a significant role in hearing or balance until recently. Hearing and balance are controlled primarily by the vestibulocochlear nerve (CN VIII), which contains special sensory and parasympathetic fibers [15]. Sympathetic innervation to the inner ear is provided by a vascular plexus on the labyrinthine artery [15]. Adrenergic receptors are also present in the inner ear, indicating sympathetic neurotransmitter action [14]. Sympathetic hyperactivity is hypothesized to be mediated at least partly through psychogenic stress [15].

Red ear syndrome, in contrast, is hypothesized to result from parasympathetic hyperactivity/sympathetic inhibition, leading to vasodilation [16]. Ipsilateral facial flushing has been observed to be more pronounced in some patients with sympathetic denervation [17]. Sympathetic innervation in the pinna is from sympathetic fibers traveling in the greater auricular nerve (C2-3) from the superior cervical ganglion, and parasympathetic innervation from the auricular branch of the vagus nerve (CN X) [15].

This patient may have autonomic dysregulation in the right facial region, resulting in alternating hyperactivity and hypoactivity of parasympathetic fibers (with relative sympathetic inhibition and excitation). Opposing mechanisms would explain why the symptoms of Ménière’s disease (sympathetic hyperactivity) and red ear syndrome (parasympathetic hyperactivity) have not occurred at the same time. An etiology for parasympathetic dysfunction is not apparent but could cause both sets of symptoms.

Increased vascular permeability

Increased vascular permeability in the cochlea has been reported in patients with Ménière's disease [14]. It was hypothesized to be due to sympathetic hyperactivity, which could lead to oxidative stress and damage to the vessels of the inner ear [14]. Sympathetic activity typically causes vasoconstriction and decreased vascular permeability, but chronic inflammation driven by oxidative stress, including the effects of IL-6, IL-1β, and TNF-α, can increase vascular permeability over time [14]. Skin biopsies for red ear syndrome have revealed superficial perivascular lymphocytic infiltrate, which is often a sign of increased vascular permeability [16]. Increased vascular permeability in the ear could also be caused by chronic inflammation over time.

Histamine, an inflammatory mediator, causes vasodilation in the peripheral nervous system and vasoconstriction in the central nervous system [18]. A chronic inflammatory condition, in which the central nervous system’s blood supply to inner ear structures experiences vasoconstriction while the peripheral nervous system’s blood supply to external ear structures experiences vasodilation, could explain the co-occurrence of Ménière's disease and red ear syndrome. However, it’s unclear why the symptoms would be unilateral rather than bilateral, as one might expect with systemic inflammation. The patient declined a skin biopsy, so the presence of inflammatory markers is unclear.

Hormonal associations

Elevated vasopressin (anti-diuretic hormone {ADH}) was one of the early explanations for Ménière’s symptoms after endolymphatic hydrops was discovered, due to the fluid imbalance inherent within the diagnosis [19]. Further research has supported that elevated plasma vasopressin exists during Ménière’s attacks, and patients may also demonstrate increased type-2 receptors for vasopressin in the endolymphatic sac, upregulated cyclic adenosine monophosphate (cAMP) activity, and increased cAMP sensitivity to vasopressin [19]. These factors could explain increased fluid pressure in the endolymphatic sac, but it's unclear why they would be dysregulated.

Psychogenic stress has been linked to increases in both vasopressin and cortisol, which have a symbiotic relationship [20]. The patient noted moderate stress, and stress does noticeably increase the frequency and severity of her symptoms. Episodic facial flushing can also be due to increased cortisol levels [20]. As facial flushing can occur in the face and ears, and red ear syndrome can occur in the ears and face, albeit unilaterally, cortisol may be a mediating factor that has not been explored in red ear syndrome. Its unilateral presentation in this patient may indicate concurrent influence by autonomic factors.

Limitations

As an individual case study of a co-occurrence of Ménière’s disease and red ear syndrome, no definitive pathophysiologic pathway can be identified with the subjective and objective information available. Both Ménière’s disease and red ear syndrome are thought to have varied and multiple etiologies, and the medical community's understanding of them is still limited and insufficient.

## Conclusions

Several hypotheses based on anatomical pathophysiology have been proposed, which could link Ménière’s disease and red ear syndrome in this 43-year-old female. Migraine, autonomic dysfunction, vascular permeability, and the cortisol-vasopressin relationship also have overlapping characteristics and pathophysiology, further showcasing the complex etiologies of these neurovascular symptoms. A final hypothesis is that the concurrent Ménière’s disease and red ear syndrome are unrelated in this patient, a hypothesis that may be supported by the lack of other literature on their co-occurrence. However, as red ear syndrome is benign and may cause only minimal pain in some patients, it’s possible that a link between Ménière’s disease and red ear syndrome has been missed or dismissed as clinically irrelevant. Given the unknowns that continue to plague patients and researchers regarding this multitude of potentially related syndromes, further research will be beneficial for clarifying potential subtypes and for establishing gold-standard diagnosis and treatment for each.
